# Xylem Sap Proteome Analysis Provides Insight into Root–Shoot Communication in Response to flg22

**DOI:** 10.3390/plants13141983

**Published:** 2024-07-20

**Authors:** Romana Kopecká, Martin Černý

**Affiliations:** Department of Molecular Biology and Radiobiology, Faculty of AgriSciences, Mendel University in Brno, 61300 Brno, Czech Republic

**Keywords:** proteomics, exudates, biotic interaction, barley, potato, protein extraction, HSP70

## Abstract

Xylem sap proteomics provides crucial insights into plant defense and root-to-shoot communication. This study highlights the sensitivity and reproducibility of xylem sap proteome analyses, using a single plant per sample to track over 3000 proteins in two model crop plants, *Solanum tuberosum* and *Hordeum vulgare*. By analyzing the flg22 response, we identified immune response components not detectable through root or shoot analyses. Notably, we discovered previously unknown elements of the plant immune system, including calcium/calmodulin-dependent kinases and G-type lectin receptor kinases. Despite similarities in the metabolic pathways identified in the xylem sap of both plants, the flg22 response differed significantly: *S. tuberosum* exhibited 78 differentially abundant proteins, whereas *H. vulgare* had over 450. However, an evolutionarily conserved overlap in the flg22 response proteins was evident, particularly in the CAZymes and lipid metabolism pathways, where lipid transfer proteins and lipases showed a similar response to flg22. Additionally, many proteins without conserved signal sequences for extracellular targeting were found, such as members of the HSP70 family. Interestingly, the HSP70 response to flg22 was specific to the xylem sap proteome, suggesting a unique regulatory role in the extracellular space similar to that reported in mammalians.

## 1. Introduction

The plant vasculature serves as a vital lifeline, transporting energy-rich molecules, building blocks, and nutrients to various organs while providing structural support [1]. It also functions as an efficient long-distance communication network, relaying information about environment cues both above and below ground [2]. Molecular and physiological aspects, including electrical signals, hydraulic pressure changes, carbohydrate and hormonal signaling, calcium (Ca^2+^) and reactive oxygen species (ROS) waves, assimilate transport, signaling peptides, RNA, and proteins, have been detected in the vasculature, ensuring optimal plant functioning in constantly changing environments [3,4,5,6,7,8,9]. This intricate system comprises two specialized transport channels: phloem and xylem. The phloem primarily translocates carbohydrates from sources to sinks but also plays a critical role in long-distance signaling and defense by transporting hormones, RNA, and proteins [10,11,12,13]. The xylem tissue of angiosperms is distinguished by its intricate arrangement of vertically oriented, multicellular vessel elements that are highly efficient and provide low-resistance water transport [14]. Primarily responsible for transporting water and minerals from roots to shoots, it also plays a significant role in root-to-shoot communication [15]. The long-distance coordination between shoots and roots is fundamental for optimal growth, development, and survival of vascular plants [16]. One of the best-characterized long-distance signal transduction pathways involves phytohormones. Xylem sap has been shown to transport various compounds, including trans-zeatin-like cytokinins, abscisic acid, strigolactones, the ethylene precursor 1-aminocyclopropane-1-carboxylic acid, gibberellins, methyl jasmonate, and methyl salicylate. On the other hand, phloem sap transports isopentenyl-type cytokinins, abscisic acid, methyl jasmonate, and auxins [9,16,17,18,19,20,21,22,23].

Proteins are a major component of both phloem and xylem saps. This has fueled the emergence of vascular sap proteomics, a field that has witnessed significant progress in the past two decades thanks to advancements in technology. Early studies revealed that long-distance protein movement in the phloem involves both nonselective bulk flow and a regulated process mediated by protein–protein interactions within sieve tubes [24], and it was proposed that proteins involved in plant signaling might be produced in the roots and travel long distances to the shoots [25]. The discovery of phloem proteins involved in the systemic acquired resistance (SAR) response spurred further interest in sap proteomics [26]. Recent advancements in molecular techniques have significantly enhanced our understanding of the plant vasculature’s role in plant–pathogen interactions. Various analyses of xylem sap during plant–pathogen interactions have been conducted on species such as *Brassica oleracea*, *Glycine max*, *Brassica napus*, *Solanum lycopersicum*, and *Vitis vinifera*, revealing the accumulation of pathogenesis-related (PR) and redox-related proteins, suggesting their protective roles in plant vasculature [27,28,29,30,31]. The increased sensitivity of new techniques has led to the detection of a wider range of proteins in xylem sap. Numerous proteins induced in response to abiotic and biotic stressors have been identified, including defense-related PR proteins, β-1,3-glucanases, chitinases, endochitinases, and proteases [9,27,32,33,34].

Pathogen infection can significantly alter the protein composition of xylem sap, and xylem can also be colonized by viruses, bacteria, and fungi [27,35]. Plants deploy a robust immune system that relies on their ability to recognize invading microbes. When bacteria enter plant tissues, the receptor FLS2 detects a short, conserved amino acid sequence (flg22) within the bacterial flagellin protein. This recognition triggers the activation of plant immunity and the FLS2 receptor is also needed for flg22 delivery to vascular tissue and its long-distance transport [36]. This mechanism is evolutionarily conserved, but it is not the sole means of bacterial flagellin perception [37]. Multiple studies have focused on flagellin because of its ability to induce the expression of defense-related genes and trigger resistance to pathogenic bacteria. However, despite numerous successes in uncovering molecular mechanisms behind its action [38,39], our knowledge is far from complete.

The research presented here investigates a faster and more sensitive method for analyzing xylem sap protein composition. We evaluated the reliability and reproducibility of shorter sampling intervals using a single plant per sample. Our findings confirm the feasibility of this approach and reveal novel insights into plant responses to biotic stimuli.

## 2. Results

**Figure 1:**
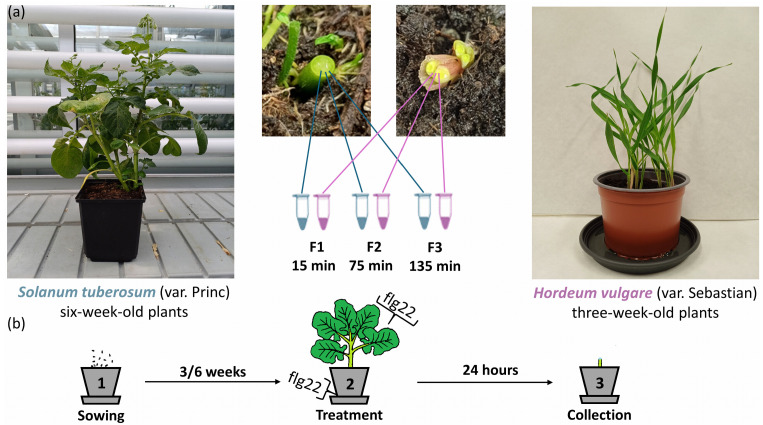
Experimental design. (**a**) Stems of plants grown in soil were cut, and the xylem sap was collected in three consecutive fractions (15–135 min). Representative images of *S. tuberosum* (**left**) and *H. vulgare* (**right**) plants used in the experiment; (**b**) a subset of plants was pre-incubated with mock (water) or flg22 applied by spraying the leaves and pouring the solution under the pot as described in Materials and Methods. After 24 h, plants were cut, and the third fraction of the sap (75–135 min) was collected. Each experiment included at least three independent biological replicates.

While numerous experiments have successfully demonstrated the importance of apoplastic signaling in plants, established methods for analyzing the apoplastic proteome are often laborious and time-consuming, requiring large numbers of plants. These limitations can hinder both the reproducibility and reliability of the results. In this study, we employed proteomics, as described in the Section 4 and depicted in Figure 1, to analyze the xylem sap proteome of two model crops, *Solanum tuberosum* and *Hordeum vulgare*.

### 2.1. Majority of the Identified S. tuberosum Proteins in Xylem Sap Is Predicted to Be Extracellular

**Figure 2:**
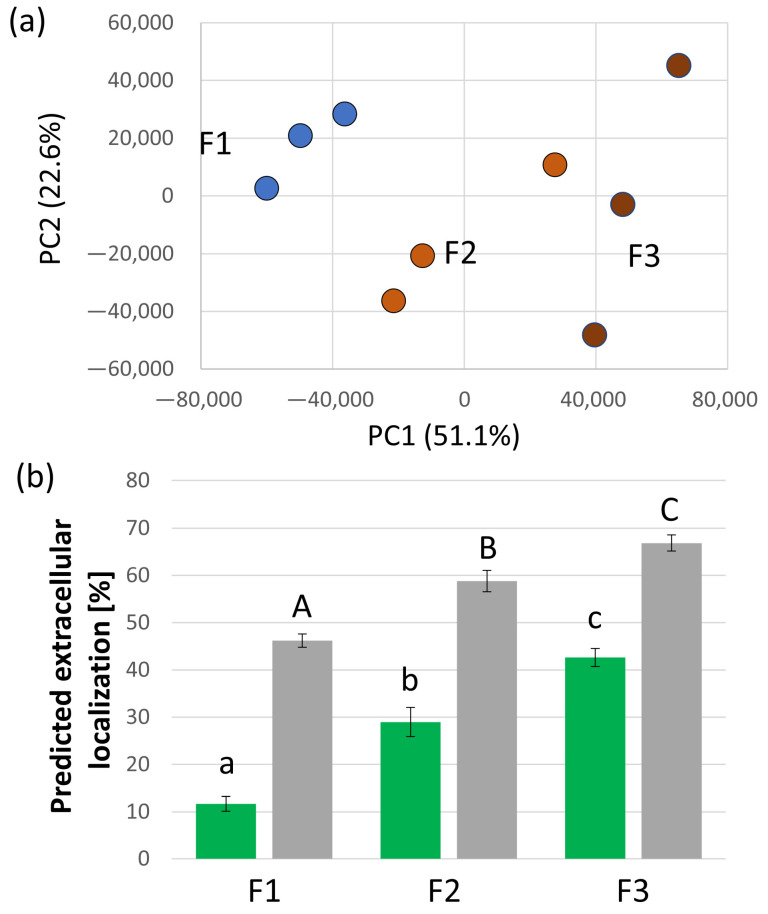
Proteome analysis of *S. tuberosum* sap. (**a**) Principal component analysis (PCA) of quantified proteins in three consecutive fractions (F1–F3). Results are based on three biological replicates. (**b**) Proportion of the proteome extract formed by proteins predicted to have extracellular localization. Proteins were assigned based on homology to *Arabidopsis* orthologs in the SUBA database (green; SUBA database [40]) and predictions for *S. tuberosum* proteins from the cropPAL database (gray; cropPAL database [41]). The plots represent the means and standard deviations of three biological replicates. Different letters indicate significant differences (ANOVA, Tukey’s HSD, *p* < 0.05). See Supplementary Table S1 for details.

To estimate the contamination level and separate putative contaminants from the true components of xylem, we collected three consecutive fractions, reasoning that the proteins rapidly depleted most likely originated from damaged tissue and did not represent the real xylem proteome. Altogether, 918 proteins, representing 13.4% of the estimated total protein abundance in the first fraction, were significantly less abundant in the subsequent two fractions, and the differences between the fractions were also clearly visible in PCA (Figure 2a). The expected localization of the closest *Arabidopsis* orthologs showed that the majority of these proteins were not expected to be extracellular, with only 23 putative extracellular proteins, indicating a possible false negative rate of 2.5%. Next, we searched for all predicted localizations of identified proteins and their *Arabidopsis* orthologs. In total, 1170 and 487 proteins are predicted to be extracellular based on the cropPAL (any predictor) and SUBA (SUBAcon algorithm) databases, respectively. As illustrated in Figure 2b, these proteins constituted less than 50% of the first collected fraction and almost 70% of the third one, representing a 1.4-fold enrichment. Interestingly, the orthologs of *Arabidopsis* extracellular proteins that contributed less than 12% to the estimated protein abundance in the first fraction were enriched almost four-fold. This corroborated our findings, showing that the third fraction is the most suitable for analyzing extracellular proteins originating from the xylem sap. One could consider collecting additional fractions. However, the destructive nature of xylem sap collection can impact plant proteosynthesis and signaling. Therefore, the collection time must be limited to minimize experimental bias.

### 2.2. Functional Diversity of Putative Extracellular Xylem Sap Proteins in Solanum tuberosum

**Figure 3:**
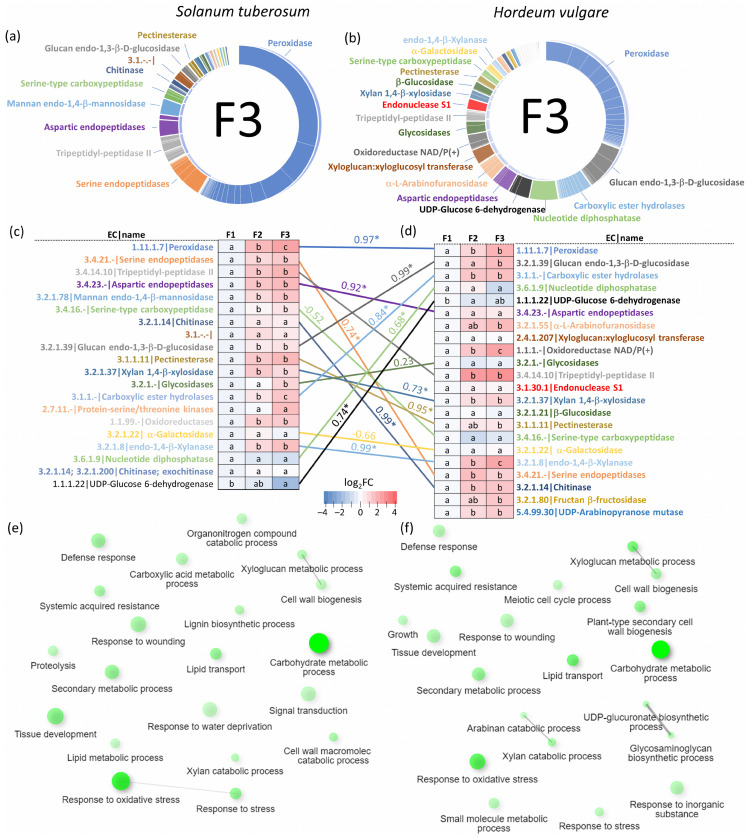
Sap proteome composition of *Solanum tuberosum* (**left**) and *Hordeum vulgare* (**right**). (**a**,**b**) Estimated abundances of identified enzymes in the third collected fraction; (**c**,**d**) differences in categories representing ≥95% of the total identified enzyme abundances visualized on a heat map. The letters represent significant differences (*p* < 0.05, ANOVA, Tukey’s HSD), the numbers above the connecting lines represent Pearson’s correlation coefficient, and statistically significant correlations (*p* < 0.05) are marked with asterisks; (**e**,**f**) gene ontology enrichment analyses-based annotations of identified Arabidopsis orthologs. Nodes represent enriched GO pathways, with size indicating the number of proteins associated with the pathway and color intensity reflecting enrichment significance. Pathways connected by lines share ≥20% of their protein components. Analyses were performed using ShinyGO 0.8 [42].

The analysis of proteins predicted to be extracellular revealed a diverse array of enzymes and components involved in cell wall dynamics and biotic interactions. These included expansins (facilitating cell wall loosening), endochitinases (defense against fungal pathogens), xyloglucan endotransglucosylases/hydrolases (cell wall construction), α-galactosidases and α-L-arabinofuranosidases (cell wall dissolution), β-glucosidases (potentially involved in cell wall breakdown), cellulases (degrading cellulose, a major plant cell wall component), lipid-transfer proteins (potentially influencing wax or cutin deposition in cell walls), defensins, peroxidases (involved in stress response and secondary metabolism), pathogenesis-related proteins, proteases, and protease inhibitors. Notably, the identified extracellular proteome comprised over 450 enzymes belonging to 54 distinct classes. Peroxidases (EC 1.11.1.7) constituted the most abundant fraction (exceeding 50% of estimated protein enzyme abundance), followed by serine endopeptidases (EC 3.4.21.-), tripeptidyl-peptidases II (EC 3.4.14.10), aspartic endopeptidases (EC 3.4.23.-), and mannan endo-1,4-β-mannosidases (EC 3.2.1.78) (Figure 3a). These enzymes also displayed significantly higher abundance in the second and third collected fractions (Figure 3c). Subsequent gene ontology (GO) analysis of the putative extracellular proteins revealed significant enrichment in categories related to carbohydrate metabolic processes and response to oxidative stress. Additionally, GO analysis identified categories of interest such as defense response and systemic acquired resistance (Figure 3e).

### 2.3. Solanum tuberosum Sap Proteome Response to flg22

The composition of the plant sap proteome offers valuable insights into its role in defense and immunity. To validate and expand our experiment, we compared the sap proteome of plants pretreated with a flagellin fragment (flg22), a bacterial elicitor, to controls pretreated with the mock treatment. The 24 h treatment did not induce any visually discernible changes in plant morphology, as treated and control plants appeared identical. Based on the initial findings described above, the third fraction was chosen for further analysis. To account for potential bias and contamination by intracellular proteins, estimated protein abundances were normalized to the total abundance of proteins predicted to have an extracellular localization.

**Figure 4:**
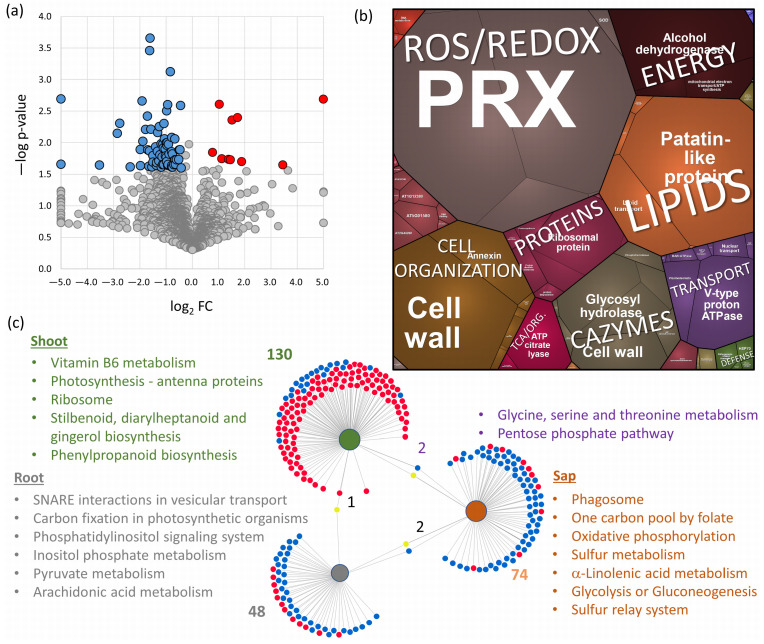
*S. tuberosum* sap proteome response to flg22. (**a**) Volcano plot representation of response to flg22 (flg22 vs. mock). Highlighted differentially abundant proteins (DAPs) represent statistically significant differences at 5% FDR. (**b**) Visualization of functional categories in the ProteoMap. The size of each category corresponds to the estimated protein abundance. (**c**) The DiVenn visualization depicts DAPs (adjusted *p*-value < 0.05, absolute fold change > 1.4) and significantly enriched metabolic pathways identified by MetaboAnalyst in DAPs specific to shoot (green), root (gray), sap proteome (orange) and in the overlap of these treatments. Red and blue dots indicate a relative increase and decrease in protein abundances compared to mock-treated control plants, respectively, while yellow dots represent differential responses between the comparisons. *S. tuberosum* proteins were annotated using the closest *Arabidopsis* orthologs. Generated using DiVenn online tool [47]. For details on identified DAPs, see Supplementary Tables S1–S4.

Analysis of the *S. tuberosum* sap proteome identified 78 differentially abundant proteins (DAPs; fold change > 1.4, adjusted *p*-value < 0.05; Figure 4a). Functional analysis revealed that the majority of these DAPs belonged to peroxidases (PRXs), lipid transport and metabolism, cell organization and wall maintenance, carbohydrate-active enzymes (CAZymes), transport, protein metabolism, and energy metabolism (Figure 4b). Notably, most of these proteins displayed a decrease in abundance following flg22 treatment. Only ten proteins exhibited an increase in abundance upon elicitor treatment. These included two peroxidases (PGSC0003DMT400082008, PGSC0003DMT400068340), a bifunctional inhibitor/lipid-transfer protein (PGSC0003DMT400044140), an ortholog of a 16 kDa phloem protein with a putative role in the defense response (PGSC0003DMT400007873), and an ortholog of a plasmodesmata callose-binding protein (PGSC0003DMT400024290) known to regulate cell-to-cell trafficking and signaling [43]. Proteins with a decrease in abundance in flg22 pretreated plants included four O-glycosyl hydrolases (PGSC0003DMT400033483, PGSC0003DMT400033482, PGSC0003DMT400000943, PGSC0003DMT400024535). These enzymes are known to play diverse roles in plant defense, potentially increasing or decreasing resistance depending on the specific pathogen [44]. Similarly, the decrease in an ortholog of zeamatin (PGSC0003DMT400009202), with putative antifungal activity [45], and a FASCICLIN-like arabinogalactan protein (PGSC0003DMT400091864), whose ortholog was recently implicated in resistance to *Plasmodiophora brassicae* [46], could suggest a shift in defense strategy in response to the perceived bacterial threat.

### 2.4. Root and Shoot Proteome Analyses Corroborate the Utility of Sap Proteome Profiling

The results of the sap proteome analyses were compared with total protein extracts obtained from the roots and shoots of collected plants. In total, 5034 and 3594 proteins were identified and quantified, respectively. The content of proteins with predicted extracellular localization represented less than 12% of the estimated total protein abundance, similar to that of the first fraction of collected xylem sap. The treatment with flg22 showed 50 DAPs in roots and 132 DAPs in shoots (Figure 4c). However, the overlap between identified DAPs in response to flg22 was negligible. Namely, a putative component of the transcriptional corepressor complex (PGSC0003DMT400060407) was significantly more abundant in shoots and significantly less abundant in roots. The comparison with DAPs identified in the sap proteome showed a similar response in the abundances of putative adenylosuccinate lyase (PGSC0003DMT400070618; root) and putative serine hydroxymethyltransferase (PGSC0003DMT400016362; shoot), and an opposite pattern for the response of glutathione peroxidase (PGSC0003DMT400012775; an increase in abundance in the roots of flg22-treated plants) and putative helicase (PGSC0003DMT400078069; an increase in abundance in the shoots of treated plants).

The functional analysis of DAPs revealed a striking tissue-specific enrichment in key metabolic pathways (Figure 4c). The shoots displayed an enrichment in proteins associated with secondary metabolism, photosynthesis, and ribosomal function. The roots exhibited a distinct enrichment of proteins involved in the pyruvate metabolism, vesicular transport, carbon fixation, and components of lipid metabolism. Interestingly, the pentose phosphate pathway and glycine/serine/threonine metabolism were jointly enriched in both the sap and shoot proteome. However, consistent with the lack of overlap in identified DAPs between different tissues, the metabolic pathways found enriched in the sap fraction were predominantly unique. These included pathways for energy metabolism, the sulfur relay system and metabolism, linolenic acid metabolism, and phagosome biogenesis (Figure 4c).

### 2.5. Xylem Sap Proteome Analysis of Hordeum Vulgare Revealed a Rich Protein Landscape and a Much Stronger Response to flg22

**Figure 5:**
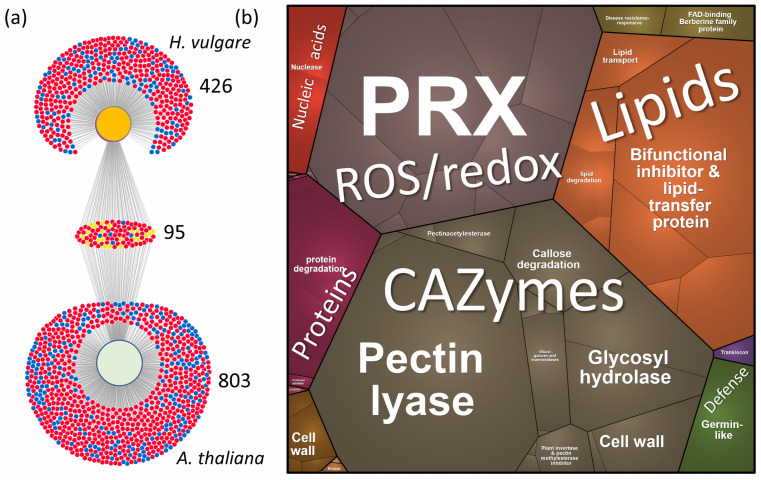
*H. vulgare* xylem sap proteome response to flg22. (**a**) The DiVenn visualization depicts DAPs (adjusted *p*-value < 0.05) found in the third collected fraction of *H. vulgare* xylem sap proteome and flg22 response proteins identified in *A. thaliana* [48]. Red and blue dots indicate a relative increase and decrease in protein abundances compared to mock-treated control plants, respectively, while yellow dots represent differential responses between the comparisons; (**b**) visualization of functional categories in the ProteoMap. Based on estimated protein abundances of DAPs that are predicted to be extracellular proteins. The size of each category corresponds to the estimated protein abundance. *H. vulgare* proteins were annotated using the closest *Arabidopsis* orthologs. For details on identified DAPs, see Supplementary Tables S5 and S6.

As detailed in the Section 4, we collected xylem sap proteomes from *H. vulgare*. The subsequent analysis yielded quantitative data for over 3300 proteins (see Supplementary Tables S2 and S5). The predicted localization of the best-matching *Arabidopsis* orthologs indicated a lower abundance of putative extracellular proteins, comprising at most 26% of the total protein content in the collected fractions. This likely contributes to a more diverse proteome composition (Figure 3b). Peroxidases were the dominant enzyme class in the dataset. However, their total abundance was only half of that observed in *S. tuberosum* (Figure 3b). Serine proteases and tripeptidyl-peptidases exhibited significantly lower abundance, whereas CAZymes showed an increase. These discrepancies might be attributed to differing developmental stages, as the *H. vulgare* plants were only three weeks old. Notably, the majority of enzyme classes displayed a significantly higher abundance in the second and third collected fractions, with the pattern generally mirroring that of *S. tuberosum* (Figure 3d). Additionally, gene ontology enrichment analysis of all putative extracellular proteins revealed substantial overlap in identified categories (Figure 3f). Next, the response to flg22 was compared in the third collected fraction using the estimated content of annotated extracellular proteins as a normalization factor. In contrast to *S. tuberosum* sap proteome, *H. vulgare* displayed a much stronger response to flg22 treatment. Over 14% of its xylem sap proteome was significantly affected, with 358 and 117 proteins showing increased and decreased abundances, respectively (fold change > 1.4, adj. *p*-value < 0.05). This suggests a more substantial reprogramming of cellular processes in barley upon exposure to flg22. To assess the broader relevance of our findings, we compared our results with a recently published dataset on the *Arabidopsis* seedling response to flg22 [48]. This analysis revealed an overlap of 95 proteins responsive to flg22 in both model organisms (Figure 5a), and most of these DAPs showed a similar response to flg22, including proteins involved in protein processing and endocytosis, biosynthesis of secondary metabolites, and CAZymes (see Supplementary Tables S5 and S6 for details). While this comparison is limited by the use of different targeted tissues, it provides an independent validation for a subset of the putative flg22-responsive proteins identified in our study.

We next investigated the functional roles of the identified xylem flg22 response proteins. To streamline the analysis and minimize potential bias, we focused exclusively on proteins predicted to localize to the extracellular space. The observed DAPs belong to the same metabolic pathways as those found in *S. tubeorosum*, dominated by CAZymes, lipid transfer proteins, peroxidases, and proteases (Figure 5b). The Flg22 response proteins identified in our study were involved in cell wall modification and defense, including various galactosidases, expansins, arabinofuranosidases, xylosidases, pectin acetylesterases, and pectin lyases. These proteins play a crucial role in modifying the plant cell wall, which serves as a primary barrier against pathogen invasion. Enzymes like α-galactosidase and β-galactosidase degrade complex carbohydrates, while expansins and pectin-modifying enzymes alter the structural properties of cell walls to bolster defense mechanisms [49]. Notably, pectin methylesterase (HORVU3Hr1G091360.3, HORVU5Hr1G010860.1, HORVU1Hr1G059010.1) was significantly less abundant in response to flg22. Interestingly, in *Arabidopsis*, pectin methylesterase is induced upon infection, and the corresponding mutant exhibits increased resistance to bacterial infection [50].

## 3. Discussion

Plant adaptation heavily relies on resource allocation and communication between shoots and roots, facilitated by plant vasculature through various signals, including electrical signals, hydraulic pressure changes, sugar and hormone signaling, calcium (Ca^2+^) and reactive oxygen species waves, assimilate transport, and the presence of peptides, RNA, esDNA, and proteins [19,32,51]. This dynamic communication ensures optimal plant functioning in an ever-changing environment. Several techniques have been established for extracting plant extracellular proteins, facilitating the analysis of processes occurring in the apoplast. Cell culture methods offer a comprehensive view of secreted proteins (e.g., [52]). However, these approaches lack the ability to investigate the role of the extracellular proteome in inter-tissue communication. For analyses at the tissue level, phloem sap collection can be achieved through EDTA-facilitated exudation, wound-induced exudation, or insect-assisted stylectomy (e.g., [53,54]). Apoplastic fluid can be isolated using vacuum infiltration–centrifugation techniques (reviewed in [15,26]). Xylem sap, on the other hand, is primarily collected via methods relying on root pressure or spontaneous exudation after stem cutting (reviewed in [55]). While these protocols provide valuable information, their laborious nature and requirement for large quantities of time or plant material can hinder widespread adoption.

Our analyses demonstrated that the xylem sap proteome can be effectively monitored, and sampling from a single plant for 60 min suffices to provide reproducible insights into the abundances of over 570 and 400 proteins with predicted extracellular localization in *Solanum tuberosum* and *Hordeum vulgare*, respectively. Likely, the portion of true extracellular proteins delivered through noncanonical secretory pathways [56] is much higher. We hypothesize that proteins whose abundances did not decrease in consecutive fractions are true extracellular proteins, though validating these findings will require further extensive research. The analysis of the xylem sap proteome has proven to be a valuable method for understanding plant defense mechanisms and pathogen interactions. However, the sampling protocol can be further optimized. The relatively high content of proteases suggests the inclusion of a protease inhibitor cocktail might be advantageous. We speculate that endogenous protease activity, despite the presence of inhibitor proteins and the anticipated specificity of secreted proteases, might have led to partial protein loss in our experiments. Alternatively, collecting sap into tubes containing denaturing agents, such as 10% trichloroacetic acid in acetone, could precipitate the isolated proteins and minimize residual enzymatic activity.

### 3.1. Novel flg22 Response Proteins Identified by Xylem Sap Proteome Analysis

Flagellin, a bacterial protein forming the flagellum, is also pivotal in plant immunity. Its N-terminus, represented by the peptide flg22, acts as a pathogen-associated molecular pattern (PAMP), triggering plant defense responses [57]. Studies on the effects of flagellin application on pathogen resistance have been conducted in various model plants, including *Arabidopsis*, *Oryza sativa*, *Glycine max*, *Solanum lycopersicum*, and *Nicotiana tabacum* [57,58,59,60,61]. Our xylem sap proteome analysis identified novel components of plant immunity in response to flg22 and assigned putative functions to previously unannotated proteins. These included components of signal transduction such as calcium/calmodulin-dependent serine/threonine-kinase (HORVU4Hr1G066750.1), an ortholog of G-type lectin S-receptor serine/threonine-protein kinase (HORVU3Hr1G110350.1), and signal peptidase complex subunit (HORVU5Hr1G088300.1). While these proteins have not been characterized, G-type lectin receptor kinases and calcium-dependent kinases are known to be involved in plant–microbe interactions [62] and plant immunity [63], respectively.

Proteins of interest that accumulated in response to flg22 included protease inhibitors (cysteine proteinase inhibitor HORVU3Hr1G038190.1; serpin-ZX, HORVU4Hr1G013550.2), an ortholog of protein EXORDIUM-like (HORVU6Hr1G077750.1), subtilisin protease (HORVU5Hr1G061990.1), and an endonuclease (HORVU2Hr1G112860.1). Protease inhibitors regulate proteolytic activities during pathogen attacks, inhibiting pathogen-secreted proteases and controlling endogenous proteases involved in programmed cell death to prevent excessive cell death and limit pathogen spread within plant tissues [64]. EXORDIUM regulates brassinosteroid-responsive genes and is proposed to be part of the flg22 response in *Arabidopsis* [65]. The endonuclease identified is an ortholog of *Arabidopsis* Endonuclease 2, known to play a role in biotic interactions. Finally, two subtilases were recently found as apoplastic proteases responsible for the C-terminal cleavage of flg22, and mutants lacking these subtilases displayed a decrease in ROS production in response to flg22 [66]. That suggests that HORVU5Hr1G061990.1 likely performs the same function in *H. vulgare*.

### 3.2. Response of Lipid Metabolism to flg22 Is Evolutionarily Conserved in H. vulgare and S. tuberosum

Lipid transfer proteins and lipases were among the DAPs in the xylem sap of both model plants, showing a similar response to flg22. In *H. vulgare*, four lipid transfer proteins were significantly less abundant (HORVU2Hr1G107480.2, HORVU2Hr1G107470.2, HORVU2Hr1G073730.1, HORVU1Hr1G046400.1), while *S. tuberosum* exhibited a decrease in glycolipid transfer protein (PGSC0003DMT400051703) and lipid-binding protein (PGSC0003DMT400057410). A similar pattern was observed for GDSL esterases/lipases and phospholipase patatin (HORVU2Hr1G107480.2, HORVU2Hr1G107470.2, HORVU2Hr1G073730.1, HORVU1Hr1G046400.1). These proteins modify membrane lipids, influencing membrane stability and integrity under biotic stress, which is crucial for pathogen resistance as they affect membrane permeability and signaling functions, enhancing plant defense capabilities. Notably, lipid transfer proteins can also directly target and disrupt bacterial cell membranes [67]. Beyond their role in membrane modification, lipid transfer proteins have been implicated in distal transport, potentially facilitating the movement of nutrients and signaling molecules like phytohormones [8].

### 3.3. HSP70 Family Proteins in Response to flg22 Suggests Their Extracellular Role

Despite targeting primarily proteins with a predicted extracellular localization, members of the HSP70 family were also among the DAPs in both model plants. HSP70 proteins are key components of cell maintenance and have a role in both abiotic and biotic signaling [68]. In *H. vulgare*, five HSP70 proteins were significantly more abundant in flg22-treated samples (HORVU1Hr1G030790.1, HORVU3Hr1G073230.1, HORVU4Hr1G012460.2, HORVU1Hr1G027420.2, HORVU2Hr1G112630.2), whereas *S. tuberosum* showed decreased abundance in an ortholog of cytosolic HSP70-14 (PGSC0003DMT400080581) and HSP70-interacting protein (PGSC0003DMT400038437). Interestingly, these proteins did not show significant changes in root or shoot tissues of *S. tuberosum*, implying a potential extracellular role for HSP70, as documented in mammalian systems [69].

## 4. Materials and Methods

### 4.1. Plant Material and Growth Conditions

Potato (*Solanum tuberosum*, variety Princ) and barley (*Hordeum vulgare*, variety Sebastian) plants were cultivated in a controlled environment. Plants were grown in Potgrond H soil (Klasmann-Deilmann GmbH, Geeste, Germany) under a 12 h photoperiod with a constant temperature of 21 °C and a photon flux density of 100 μmol m^−2^ s^−1^. Potato tubers were used as starting material, while barley plants were grown from surface-sterilized seeds. After six weeks of cultivation for potato and three weeks for barley, the sap proteome was collected by cutting the stems 10–20 mm above the soil surface using a razor blade. The xylem sap was sampled in three fractions over a two-hour period: F1 (15 min), F2 (75 min), and F3 (135 min). Fractions were collected every 5–10 min using a pipette, aspirating an average of 450 µL per hour for *S. tuberosum* and 50 µL per hour for *H. vulgare*. Upon collection, the sap was immediately flash-frozen in liquid nitrogen. Each experiment included at least three independent biological replicates. The experimental design is summarized in Figure 1.

### 4.2. Elicitor Treatment

A subset of plants was pre-incubated with flg22, a conserved peptide sequence derived from bacterial flagellin (QRLSTGSRINSAKDDAAGLQIA, >95% purity; ProteoGenix, Schiltigheim, France). Flg22 solution (1 μM flg22, 0.025% *v*/*v* Silwet L-77) was applied by spraying the leaves and pouring the solution under the pot. Potato plants received 30 mL and 200 mL of the solution for spraying and watering, respectively. Due to their smaller size, barley plants were treated with half the solution volume. Mock-treated plants received a solution containing only 0.025% Silwet L-77. After 24 h, plants were cut, and the third fraction of the sap (75–135 min) was collected. Root and shoot tissues were collected in liquid nitrogen, the sap was collected as described above. Each experiment included at least three independent biological replicates.

### 4.3. Proteome Analysis

The collected sap was flash-frozen and lyophilized. Next, the samples were washed with 300 µL of a 3:1 (*v*/*v*) methyl tert-butyl ether/methanol mixture and with 200 µL of 80% (*v*/*v*) acetone in water. Finally, the samples were solubilized in urea and digested using the previously described protocol [70]. The resulting peptides were desalted using Strata C18-E and concentrated to 15 µL. Portions of samples corresponding to 5 μL were analyzed by nanoflow reverse-phase liquid chromatography–mass spectrometry using a 25 cm C18 Biozen 2.6 μm Peptide XB-C18 column (Phenomenex, Torrance, CA, USA), a Dionex Ultimate 3000 RSLC nano-UPLC system, and the Orbitrap Fusion Lumos Tribrid Mass Spectrometer equipped with a FAIMS Pro Interface (Thermo Fisher, Waltham, MA, USA). All samples were analyzed using FAIMS compensation voltages of −40, −50, and −75 V, as described previously [71]. The measured spectra were recalibrated and searched against the *S. tuberosum* (SolTub_3.0–GCA_000226075) and *H. vulgare* (MorexV3_pseudomolecules_assembly–GCA_904849725.1; *Hordeum vulgare* IBSC v2) databases, and common contaminants databases using Proteome Discoverer 2.5 (Thermo Fisher Scientific) employing Sequest HT, MS Amanda 2.0 [72], or MSFragger [73] algorithms. The settings were as follows: enzyme–trypsin, max two missed cleavage sites; MS1 tolerance—5 ppm; MS2 tolerance—0.1 Da, SEQUEST/0.02 Da, MS Amanda; fixed modifications—carbamidomethyl (Cys); dynamic modifications including Met oxidation, Asn/Gln deamidation; and dynamic modifications at the end of the protein—acetylation (N-end); loss of methionine (N-terminus); loss of methionine/acetylation (N-terminus). The MSFragger was implemented using ProteomeDiscoverer and default settings for a closed search. The resulting peptide hits were filtered for a maximum 1% false discovery rate using the Percolator Node (Proteome Discoverer 2.5). Root and shoot proteomes were analyzed as described previously, e.g., [74]. The quantitative analysis centered on (i) proteins identified by two or more unique peptides and (ii) proteins with a single unique peptide but at least ten assigned peptides, aiming for broader proteome coverage. The mass spectrometric proteomic data acquired were deposited in the ProteomeXchange Consortium (http://proteomecentral.proteomexchange.org) via the PRIDE partner repository [75] with the dataset identifier PXD053406.

### 4.4. Data Analysis and Processing

The reported statistical tests were generated and implemented using the Real Statistics Resource Pack software for MS Excel (Release 6.8; Copyright 2013–2020; Charles Zaiontz; www.real-statistics.com), MetaboAnalyst 6.0 [76], SIMCA 14.1 (Sartorius), Proteome Discoverer 2.5 (Thermo Fisher Scientific), and Proteomaps [77]. Significant differences refer to *p* < 0.05, adj. *p*-value represents Benjamini and Hochberg procedure at 5% FDR.

## 5. Conclusions

Our study demonstrated that with modern proteomics’ sensitivity, xylem sap can be collected from a single plant, providing sufficient and reproducible protein yields. This approach, as evidenced by our experiments with the flg22 treatment, can offer novel insights into plant–microbe interactions. Despite providing evidence on the reproducibility of proteomics data in the collected xylem sap and demonstrating the complementarity of the results found in the roots, shoots, and xylem saps, it is crucial to acknowledge that this simple and inexpensive technique for collecting xylem sap is prone to contamination from both phloem and cellular spillage, as evidenced by the first collected fractions. Bioinformatics tools indicate that the majority of proteins identified in the third collected fraction are extracellular. However, this does not confirm their origin in the xylem. Therefore, we recommend exercising caution when using our list of identified proteins as sole evidence for their presence in the xylem.

## Data Availability

The mass spectrometric proteomic data acquired have been deposited in the Proteo-meXchange Consortium (http://proteomecentral.proteomexchange.org) via the PRIDE partner repository [75] with the dataset identifier PXD053406.

## Supplementary Materials

The following supporting information can be downloaded at: https://www.mdpi.com/article/10.3390/plants13141983/s1, Table S1: Proteins identified in *S. tuberosum* xylem sap; Table S2: Proteins identified in *H. vulgare* xylem sap; Table S3: Flg22 response proteins in *S. tuberosum* xylem sap proteome; Table S4: Flg22 response proteins in *S. tuberosum* root and shoot tissues; Table S5: Flg22 response proteins in *H. vulgare* xylem sap proteome; Table S6: Flg22 response proteins in *Arabidopsis* [49].
